# *Haemophilus influenzae* carriage and antibiotic resistance profile in Belgian infants over a three-year period (2016–2018)

**DOI:** 10.3389/fmicb.2023.1160073

**Published:** 2023-04-24

**Authors:** Esra Ekinci, Laura Willen, Juan Pablo Rodriguez Ruiz, Kirsten Maertens, Liesbet Van Heirstraeten, Gabriela Serrano, Magali Wautier, Ariane Deplano, Herman Goossens, Pierre Van Damme, Philippe Beutels, Surbhi Malhotra-Kumar, Delphine Martiny, Heidi Theeten

**Affiliations:** ^1^Centre for the Evaluation of Vaccination, University of Antwerp, Wilrijk, Belgium; ^2^Laboratory of Medical Microbiology, University of Antwerp, Wilrijk, Belgium; ^3^National Reference Centre for Haemophilus influenzae, Laboratoire Hospitalier Universitaire de Bruxelles – Universitair Laboratorium Brussel (LHUB-ULB), Brussels, Belgium; ^4^Centre for Health Economics Research and Modelling Infectious Diseases, University of Antwerp, Wilrijk, Belgium

**Keywords:** *Haemophilus influenzae*, children, biotypes, serotypes, mutations, antibiotic susceptibility testing

## Abstract

**Background:**

Non-typeable *Haemophilus influenzae* has become increasingly important as a causative agent of invasive diseases following vaccination against *H. influenzae* type b. The emergence of antibiotic resistance underscores the necessity to investigate typeable non-b carriage and non-typeable *H. influenzae* (NTHi) in children.

**Methods:**

Nasopharyngeal swab samples were taken over a three-year period (2016–2018) from 336 children (6–30 months of age) attending daycare centers (DCCs) in Belgium, and from 218 children with acute otitis media (AOM). Biotype, serotype, and antibiotic resistance of *H. influenzae* strains were determined phenotypically. Mutations in the *ftsI* gene were explored in 129 strains that were resistant or had reduced susceptibility to beta-lactam antibiotics. Results were compared with data obtained during overlapping time periods from 94 children experiencing invasive disease.

**Results:**

Overall, NTHi was most frequently present in both carriage (DCC, AOM) and invasive group. This was followed by serotype “f” (2.2%) and “e” (1.4%) in carriage, and “b” (16.0%), “f” (11.7%), and “a” (4.3%) in invasive strains. Biotype II was most prevalent in all studied groups, followed by biotype III in carriage and I in invasive strains. Strains from both groups showed highest resistance to ampicillin (26.7% in carriage vs. 18.1% in invasive group). A higher frequency of *ftsI* mutations were found in the AOM group than the DCC group (21.6 vs. 14.9% – *p* = 0.056). Even more so, the proportion of biotype III strains that carried a *ftsI* mutation was higher in AOM compared to DCC (50.0 vs. 26.3% – *p* < 0.01) and invasive group.

**Conclusion:**

In both groups, NTHi was most frequently circulating, while specific encapsulated serotypes for carriage and invasive group were found. Biotypes I, II and III were more frequently present in the carriage and invasive group. The carriage group had a higher resistance-frequency to the analyzed antibiotics than the invasive group. Interestingly, a higher degree of *ftsI* mutations was found in children with AOM compared to DCC and invasive group. This data helps understanding the *H. influenzae* carriage in Belgian children, as such information is scarce.

## Introduction

The nasopharynx is a major reservoir for bacterial pathogens that can lead to upper respiratory tract infections in children and nasopharyngeal carriage of pathogens is an important source of transmission to other susceptible groups, mainly elderly (Aniansson et al., 1992). *Haemophilus influenzae* is one of the most important bacterial colonizers of the nasopharynx and is a frequent cause of acute otitis media (AOM) and sinusitis (Hashida et al., 2008; Ortiz-Romero et al., 2017). *H. influenzae* can exist without a capsule (non-typeable *H. influenzae*) or can be capsulated by six different polysaccharide capsules (i.e., serotypes a to f; Pittman, 1931), with the most virulent one being *H. influenzae* serotype b (Hib). Up until the introduction of the *H. influenzae* vaccine — that included the Hib capsule — into the national childhood vaccination programs in 1993 (Jacquinet et al., 2021), Hib was the most common cause of invasive *H. influenzae* disease in young children, including meningitis (Pittman, 1931; Peltola et al., 1977; Peltola, 2000). The routine administration of Hib vaccines led to a significant decline in invasive Hib disease in all age groups, through direct and indirect (herd immunity) protection (Swingler et al., 2007); but this in turn made room for non-typeable *H. influenzae* (NTHi) cases as well as non-b-serotype cases to increase (Madore, 1996; Agrawal and Murphy, 2011). In Belgium, most (76.8%) of the invasive *H. influenzae* strains were reported as non-typeables during the period 2017–2021 (Grammens et al., 2017, 2018; Jacquinet et al., 2021). However, typeable serotypes, such as serotype “f” (9.7%), serotype “b” (5.8%), serotype “a” (2.7%), serotype “e” (1.1%) and serotype “d” (0.4%), were also found to be the cause of invasive infections in children and elderly during the same period (Grammens et al., 2017, 2018; Jacquinet et al., 2021). These observations demonstrate the importance of conducting further research into the circulating serotypes of *H. influenzae*.

*H. influenzae* strains can acquire antibiotic resistance by two different mechanisms; either by producing beta-lactamase enzymes which hydrolyze beta-lactam antibiotics (Medeiros et al., 1986; McElligott et al., 2020) or by acquiring mutations in the genes encoding for penicillin-binding protein (PBP) protein (Ubukata et al., 2001, 2002). Mutations in the *ftsI* gene, which encodes PBP3, lowers the binding affinity of beta-lactam antibiotics to PBP3 (Mendelman et al., 1987); hence, the diversity of mutations in the *ftsI* gene lead to several profiles that can affect differently the beta-lactam antibiotics, making macrolides and quinolones more important alternative treatment strategies (Tanaka et al., 2019). Beta-lactamase-negative ampicillin resistant *H. influenzae* or BLNAR is one of the problems for the treatment of *H. influenzae* with beta-lactam antibiotics. Specific amino acid substitutions have been identified by sequencing of the *ftsI* gene, producing a BLNAR phenotype (Yalçın et al., 2022). BLNARS are divided into groups I, II, III and III-like defined by the amino acid mutation patterns at specific sites (Hotomi et al., 2007). Group III and III-like (referred as high-BLNAR) have usually a higher ampicillin MIC (higher level of ampicillin resistant) compared to group I and II (Han et al., 2019). High BLNARs are changing worldwide, but more rapidly in Asia than in Europe and USA (Ubukata et al., 2001; Hasegawa et al., 2003; García-Cobos et al., 2007; Barbosa et al., 2011; Skaare et al., 2014; Honda et al., 2018).

The existence of antibiotic resistance of *H. influenzae* underscores the necessity to investigate typeable non-b carriage and non-typeable *H. influenzae* in children. This study describes for the first time the carriage and antimicrobial susceptibility profile of *H. influenzae* strains in samples obtained from healthy Belgian children attending daycare centers (DCC) and children with acute otitis media (AOM), and compares it to Belgian children experiencing invasive disease caused by both non-typeable and encapsulated *H. influenzae*.

## Materials and methods

### Ethical statement

The study protocol, the informed consent forms, questionnaires and sample collection were approved by the ethics committee of University of Antwerp (UAntwerpen) and the Antwerp University Hospital (UZA; ID 15/45/471 and ID 18/31/355). Written informed consent was obtained from the infants’ parents or legal representatives.

### Study population

*Haemophilus influenzae* carriage strains were collected from children sampled in the ongoing Belgian nasopharyngeal carriage study (Np carriage study) during the sample collection periods 2016–2018 (Wouters et al., 2018; Ekinci et al., 2022). Samples were either taken from healthy children attending randomly selected DCCs or from children presenting with AOM at a physician taking part in the study (convenience sample, yearly average of 17 centers geographically equally spread across Belgium). The sample collection period covered October to May/June for children with AOM and November to March for children in DCC (except for the baseline collection between March–June in 2016). Background characteristics (including demographics, antibiotic use, and clinical data on AOM if applicable) from the NP carriage study (Wouters et al., 2018; Ekinci et al., 2022). No data was present on Hib vaccination status or date for any of the included children.

A total number of 554 strains, collected between 2016 and 2018 for the NP carriage study, were analyzed. These strains include a stratified selection of strains from children attending DCC (n = 336) and all *H. influenzae* strains present from children suffering from AOM (n = 218), from the same collection period. To make an adequate comparison between samples taken from healthy children attending DCCs and AOM-children, samples were stratified on age, sex, vaccination status and AB treatment within the two groups were selected. The selected children aged between 6 to 30 months.

Microbiological results for all invasive strains (CSF, blood) isolated in Belgian children under five between 2015 and 2018 (referred to as the “invasive group”) were provided by the Belgian National Reference Centre (NRC) for *Haemophilus influenzae* including biotype, serotype, *ftsI* sequencing (when performed), AST to ampicillin, amoxicillin-clavulanic-acid, cefotaxime and ciprofloxacin.

### Sampling and sample processing

Parents or legal representatives were asked to fill out a questionnaire before sampling. A single nasopharyngeal (NP) swab was taken with a flocked nylon swab and transported in 1 ml STGG (Skim milk – Tryptone – Glucose – Glycerol) at 2–8° C and cultured or stored at-80° C within 24 h.

### Microbiological analysis of carriage strains

Analyses were performed at the Belgian National Reference Center (NRC) for *Haemophilus influenzae*. Before testing, strains were subcultured twice onto chocolate agar (in-house preparation).

#### Identification and typing of *Haemophilus influenzae* strains

All isolates were identified by MALDI-TOF mass spectrometry using a Microflex LT spectrometer and the Biotyper 4.2.80 database (Bruker Daltonics, Bremen, Gerrmany). Biotypes and serotypes of *H. influenzae* strains were determined using biochemical tests based on the production of indole, urease and ornithine decarboxylase (Diatabs, Rosco Diagnostica, Albertslund, Denmark) and Difco Haemophilus antiserum agglutination kit (Becton Dickinson, Erembodegem, Belgium), respectively, and following manufacturer’s instructions.

#### Determination of antimicrobial resistance to beta-lactam antibiotics

Antimicrobial susceptibility testing were performed following EUCAST V9.0 guidelines and clinical breakpoints. A disk diffusion test with benzylpenicillin 1 unit disk and a beta-lactamase detection using BBL paper disks (Becton Dickinson) were performed in parallel with the susceptibility testing for ampicillin, amoxicillin-clavulanic acid and cefuroxime; ciprofloxacin was also tested. All minimum inhibitory concentrations (MIC) were determined by gradient strips antimicrobial susceptibility testing (E-test, bioMérieux, Marcy l’Etoile, France).

### Sequencing of the *ftsI* gene

The transpeptidase domain of the *ftsI* gene was sequenced as described by Ubukata et al. (2001) for all strains showing lowered susceptibilities to beta-lactams (MIC ampicillin > = 1 μg/ml combined with a negative beta-lactamase test, MIC amoxicillin-clavulanic acid > = 2 μg/ml, resistance to cefuroxime). Briefly, a 621 bp fragment is amplified by PCR and sequenced. Sequences are compared to the “wild-type” reference isolate Rd. KW20 (ATCC ® 51907) sequence (NCBI L42023.1). The amino acids substitutions are detected and listed using the BioNumerics™ software 7.6.2 (bioMérieux). BLNAR strains were classified into three groups based on amino acid substitutions: in group I (substitution in His 517 to Arg-517), in group II (substitution Lys-526 to Asn-526), and group III (substitutions in Met-377 to Ile, Ser-385 to Thr, and Leu-389 to Phe; Ubukata et al., 2001).

### Statistical analysis

Sample size was based on the number of *H. influenzae* strains obtained from children with AOM during the period 2016–2018 (*n* = 218). Similar numbers (*n* = 336) of *H. influenzae* strains obtained from children attending DCC during the period 2016–2018 were selected.

The Chi-Square (Chi^2^) Test was used to assess significant differences between different analyzed groups (α = 0.05). On serotype level Chi^2^ Test is performed on NTHi versus encapsulated *H. influenzae*. On biotype level Chi^2^ Test is performed on (in our study) frequently circulating biotypes (biotypes I, II, III) versus less frequently circulating biotypes (biotype IV, V, VI, VII).

## Results

### Characteristics of the children

The carriage group consisted of 554 samples divided in 336 samples obtained from healthy children attending DCC and 218 samples obtained from children suffering from AOM (a non-invasive disease). Children are defined as healthy if they either do not have a disease condition or they have a disease that is fully controlled which allows the child to attend the DCC. Results from the carriage groups are compared to the “invasive” group, consisting of 94 strains.

### Typing of *Haemophilus influenzae* strains

**Figure 1:**
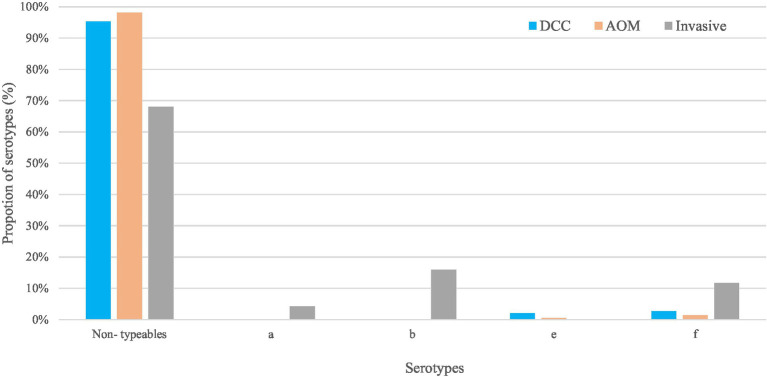
Non-typeable and typeable of *Haemophilus influenzae* in the carriage (AOM and DCC) and invasive group. Overall proportions of circulating serotypes in the carriage groups AOM (in orange) and DCC (in blue) as well as in the invasive group (in grey) detected in strains collected during the period 2016–2018 for the carriage group and 2015–2018 for the invasive group.

Non-typeable *H. influenzae* was most prevalent in both carriage (96.4%) and invasive strains (68.1%; Table 1). In the carriage group, also serotype “f” (2.2%) and serotype “e” (1.8%) were present, while in the invasive group, serotype “b” (16.0%), serotype “f” (11.7%), and serotype “a” (4.3%) were found (Table 1; Figure 1). The distribution of the different serotypes was similar between AOM and DCC children and was also similar across the different seasons (Supplementary Figure 1). The proportion of typeable serotypes was significantly higher in the invasive group compared to the carriage group (Table 1; Figure 1).

**Table 1:** Characteristics of *Haemophilus influenzae* strains in the carriage (AOM and DCC) and invasive group.

		Carriage			Invasive	Total carriage vs. invasive
		AOM *n* = 218 (*n*; %)	DCC *n* = 336 (*n*; %)	Total *n* = 554 (*n*; %)	Total *n* = 94 (*n*; %)	Chi^2^ *p*-value
Season*	2015	NA	NA	NA	24; 25.5%	
	2016	14; 6.4%	101; 30.1%	115; 20.8%	18; 19.1%	
	2017	64; 29.4%	97; 28.9%	161; 29.1%	21; 22.3%	
	2018	140; 64.2%	138; 41.1%	278; 50.2%	31; 32.9%	
Biotype	I	31; 14.2%	54; 16.1%	85; 15.3%	26; 27.7%	
	II	93; 42.6%	123; 36.6%	216; 39.0%	34; 36.2%	
	III	58; 26.6%	76; 22.6%	134; 24.2%	18; 19.1%	
	IV	5; 2.3%	7; 2.1%	12; 2.2%	2; 2.1%	
	V	26; 11.9%	49; 14.6%	75; 13.5%	11; 11.7%	
	VI	5; 2.3%	26; 7.7%	31; 5.6%	3; 3.2%	
	VII	0; 0.0%	1; 0.3%	1; 0.2%	0; 0.0%	
Biotypes	I, II, III	182; 83.5%	253; 75.3%	435; 78.5%	78; 83.0%	*p* = 0.32
	IV, V, VI, VII	36; 16.5%	83; 24.7%	119; 21.5%	16; 17.0%		NTHi	214; 98.2%	320; 95.2%	534; 96.4%	64; 68.1%	***p* < 0.001**
Typeables	4; 1.8%	16; 4.8%	20; 3.6%	30; 31.9%								
Serotype	f	3; 1.4%	9; 2.7%	12; 2.2%	11; 11.7%	
	e	1; 0.5%	7; 2.1%	8; 1.4%	0; 0.0%	
	a	0; 0.0%	0; 0.0%	0; 0.0%	4; 4.3%	
	b	0; 0.0%	0; 0.0%	0; 0.0%	15; 16.0%	
*β*-LAC	negative	167; 76.6%	255; 75.9%	422; 76.2%	79; 84.0%	*p* = 0.07
	positive	51; 23.4%	81; 24.1%	132; 23.8%	15; 16.0%	
AMP	S	161; 73.8%	245; 72.9%	406; 73.3%	77; 81.9%	*p* = 0.07
	R	57; 26.1%	91; 27.1%	148; 26.7%	17; 18.1%	
AMC	S	199; 91.3%	315; 93.8%	514; 92.8%	92; 97.9%	*p* = 0.06
	R	19; 8.7%	21; 6.3%	40; 7.2%	2; 2.1%	
CEFUR	S	141; 64.7%	224; 66.7%	365; 65.9%	NA	
	I	45; 20.6%	73; 21.7%	118; 21.3%	NA	
	R	32; 14.6%	39; 11.6%	71; 12.8%	NA	
CIP	S	217; 99.5%	335; 99.7%	552; 99.6%	93; 98.9%	*p* = 0.35
	R	1; 0.5%	1; 0.3%	2; 0.4%	1; 1.1%	
Mutation	*ftsI* gene	50; 22.9%	54; 16.1%	104; 18.8%	4; 4.3%	

*n* = number of children; Carriage = AOM and DCC children taken together; AOM = acute otitis media; DCC = daycare center; β-LAC = beta-lactamases; NTHi = non-typeable; AMP = ampicillin; AMC = amoxicillin-clavulanic acid; CEFUR = cefuroxime; CIP = ciprofloxacin; S = susceptible; I = intermediate; R = resistance; NA = not available.

* Season 2015: is only applicable for the invasive group; season 2016: May–June 2016 for carriage group, January–December 2016 for invasive group; season 2017: November 2016–March 2017 for carriage group, January–December 2017 for invasive group; season 2018: November 2017–March 2018, January–December 2018 for invasive group.

**Figure 2:**
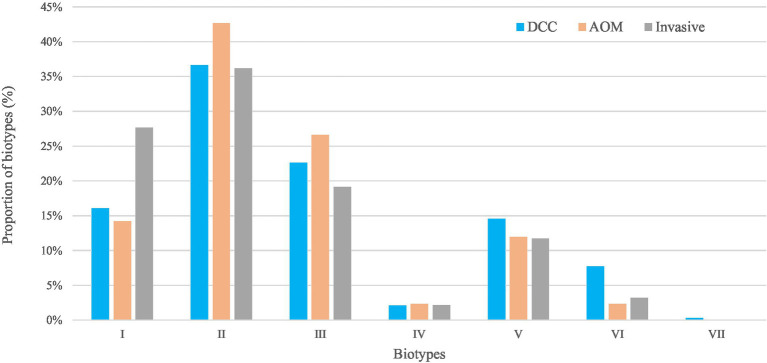
Biotypes of *Haemophilus influenzae* in the carriage (AOM and DCC) and invasive group. Overall proportions of circulating biotypes in the carriage groups AOM (in orange) and DCC (in blue) as well as in the invasive group (in grey) detected in strains collected during the period 2016–2018 for the carriage group and 2015–2018 for the invasive group.

For the carriage group, the most frequent biotypes were biotype II (39.0%), biotype III (24.2%), and biotype I (15.3%; Table 1; Figure 2). The distribution of the different biotypes was similar in the DCC and AOM population of the carriage group (Table 1) and did not differ across the different seasons (Supplementary Figure 2). For the invasive group, biotype II (36.2%), biotype I (27.7%), and biotype III (19.1%) were more frequently present (Table 1; Figure 2).

### Beta-lactam and antimicrobial resistance of *Haemophilus influenzae* strains

*H. influenzae* strains from the carriage group showed resistance to ampicillin (26.7%), cefuroxime (12.8%), amoxicillin-clavulanic acid (7.2%), and ciprofloxacin (0.4%). No difference was observed in resistance of *H. influenzae* strains across the different seasons, nor between the AOM and DCC group. Also for the invasive group, resistance was found to ampicillin (18.1%), amoxicillin-clavulanic acid (2.1%), and ciprofloxacin (1.1%). No data was provided for cefuroxime in the invasive group (not systematically tested at that time by the NRC).

Beta-lactamases were present in 23.8 and 16.0% of the *H. influenzae* strains in the carriage and invasive group, respectively (Table 1). In the carriage (*n* = 132) as well as the invasive group (*n* = 15), all of the beta-lactamase positive strains showed resistance to ampicillin, while 3.0% (4/132) and 6.7% (1/15) of the strains were resistant to amoxicillin-clavulanic acid in the carriage and invasive group, respectively. Resistance to cefuroxime was found in 10.6% (14/132) of the beta-lactamase positive strains in the carriage group.

### Mutations in the *ftsI* gene

From the 554 samples from the carriage group, 129 resistant or “less” susceptible strains were reanalyzed and were checked on mutations in the *ftsI* gene. In 81.0% (104/129) of the strains, a mutation in the *ftsI* gene was found. No difference was observed in the incidence of the *ftsI* mutation over the different time periods. Of all *ftsI* mutated strains, 93.7% corresponded to the Ubukata-group 2 and a small proportion (3.1%) to the Ubukata-group 3. A higher frequency of *ftsI* mutations was found in the strains of children with AOM compared to children attending DCCs (21.6 vs. 14.9% – *p* = 0.056). Moreover, a higher proportion of biotype III strains carried a mutated *ftsI* genein the strains of children with AOM compared to children attending DCCs (50.0 vs. 26.3% – *p* < 0.01), while no difference in mutation frequency in other biotypes was seen. All strains carrying a mutated *ftsI* gene were non-typeable.

The highest resistance of the mutated strains in the carriage group was seen to cefuroxime (56.7%; 59/104), followed by amoxicillin-clavulanic acid (35.6%; 37/104) and ampicillin (27.8%; 29/10).

In the invasive group, 4.3% (4/94) of the strains showed a mutation in the *ftsI* gene, all corresponding to the Ubukata-group 2. Biotype II was most frequently (75%; 3/4) present in these strains followed by biotype III (25%; 1/4). As in the carriage group, all mutated strains were not typeable. The mutated strains in the invasive group were resistant to ampicillin (50.0%; 2/4) and amoxicillin-clavulanic acid (50.0%; 2/4).

## Discussion

This three-year study describes the carriage status and antimicrobial susceptibility profile of *H. influenzae* strains in samples obtained from Belgian children attending DCCs and children with AOM and compares it to the susceptibility profile of *H. influenzae* strains responsible for invasive disease in children.

As expected, non-typeable *Haemophilus influenzae* strains were predominantly present in the different subgroups of our study. Also, the NRC reports Belgium showed that mostly non-typeables were present in invasive disease (81/103–78.6% in 2017; 96/129–74.4% in 2018; 128/162–79.0% in 2019; 62/84–73.8% in 2020; 68/87–78.0% in 2021). It is already known that invasive *H. influenzae* serotype b (Hib) strains have been replaced by non-typeable *H. influenzae* in many countries since the introduction of the Hib conjugate vaccines (Van Eldere et al., 2014; Zarei et al., 2016; Slack, 2017; Slack et al., 2021). Also serotypes “a,” “e,” and “f” have been reported in different countries as disease causing serotypes, and are also serotypes found in our study (Ladhani et al., 2012; Palaniappan et al., 2020; Slack et al., 2021; Reilly et al., 2022). In North America, an increase in proportion of serotype “a” has been observed among invasive disease cases (Tsang and Ulanova, 2017; Slack et al., 2021; Soeters et al., 2021; Ulanova, 2021). Serotype “a” is also found as possible disease causing serotype in our study as this serotype is only found in the invasive group but not in the carriage group. In Europe, on the other hand, serotype “f” became the most frequent encapsulated serotype causing invasive disease after implementing Hib conjugate vaccine, but the numbers remain relatively low (Ladhani et al., 2012; Reilly et al., 2022), whereas there is also an increasing trend in infections caused by serotype “e” (Ladhani et al., 2012; Slack et al., 2021). In both groups of the current study, serotype “f” was quite frequently present, supporting the findings in Europe, while serotype “e” is (rarely) present only in the carriage group. Also according the reports of the NRC in Belgium, serotype “f” is the most prevalent serotype (12/103–11.7% in 2017; 14/129–10.9% in 2018, 15/162–9.3% in 2019; 9/84–10.7% in 2020 and 5/87–5.8% in 2021), while serotype “e” was rarely present (3/103–2.9% in 2017 and 3/129–2.3% in 2018; not present in 2019–2021; Grammens et al., 2017, 2018; Jacquinet et al., 2021).

The most frequently found biotype in this study is biotype II, accounting for more than 35% in each analyzed group (DCC, AOM as well as the invasive group). This was followed by biotype III, biotype I and biotype V which is in line with observations from other studies (Albritton et al., 1978; Righter and Luchsinger, 1988; Watson et al., 1988). Different studies have found that biotype I is often associated with pathogenicity in children, such as severe meningitis, regardless whether the presence of biotype I occurred simultaneously with the presence of serotype “b” (Kilian, 1976; Long et al., 1983). These findings are supported by the results that we found in this study – a higher proportion of biotype I is found in the invasive group (27.7 vs. 15.3%). Biotype II and biotype III are commensal to the upper respiratory tract and colonization does usually not progress to invasive disease (Kilian, 1976; Long et al., 1983). However, biotype II and biotype III may be involved in the pathogenesis of mucosal diseases such as sinusitis and otitis media as well as in the pathogenesis of invasive disease (Kilian, 1976; Long et al., 1983). Also in our study, biotype II and III were more frequently found in the AOM (42.6 and 26.6%) group compared to the DCC (36.6 and 22.6%) and invasive group (36.2 and 19.1%). In addition, it is more commonly seen that biotypes I, II, III, and serotype “b” strains show resistance to ampicillin compared to other biotypes and non-serotype “b” strains (Jain et al., 2006). Interestingly, we found a higher mutation frequency in the *ftsI* gene in the strains obtained from the AOM group, especially higher proportion of biotype III in this mutated strains compared to the strains obtained from the DCC group. It is also seen that strains carrying PBP3 alterations are more invasive, suggesting that these alterations may be involved in adhesion and invasion mechanisms (Okabe et al., 2010; Atkins et al., 2014; Singh et al., 2016).

In the initial Np carriage study, frequent use of antibiotics (>3 treatments in 3 months) was an exclusion criterium in AOM children but not in DCC children. Children included from DCC turned out to have had more AB treatments than children included with AOM. However, AOM children may have used a broad-spectrum antibiotic. Measuring trends in the antimicrobial susceptibility of *H. influenzae* strains is gaining importance because of the increased use of existing antibiotics as well as development of novel antibiotics. To treat *H. influenzae* infections, aminopenicillins and cephalosporins are used as first choice, but there are mechanisms of resistance against these aminopenicillins (Schotte et al., 2019). As the number of ampicillin-resistant strains has increased, it was important to verify the effectiveness of cephalosporins (such as cefuroxime) to treat ampicillin-resistant *H. influenzae* infections (Deghmane et al., 2019; Wang et al., 2019; Nürnberg et al., 2021). This makes the development of novel antibiotic treatments important.

In conclusion, this study found that biotypes I, II and III are more frequently present in the carriage group as well as in the invasive group, while specific serotypes for carriage (serotype “e”) and invasive group (serotype “a” and “b”) were found. In addition, a higher antimicrobial resistance level for the analyzed antibiotics was observed in the carriage (DCC, AOM) group compared to the invasive group. In the strains of children suffering from AOM, a higher degree of mutations in the *ftsI* gene was found compared to the strains obtained from DCC children. The current study helps to lay the ground to understand the dynamics of *H. influenzae* carriage in Belgian children, as available data on remaining *H. influenzae* dynamics in a vaccinated child population (in a carriage as well as an invasive group) is scarce. This study provides an important first insight into the characteristics of circulating *H. influenzae* strains, especially in light of emerging antibiotic resistance to beta-lactams as they may have implications for the treatment of *H. influenzae* infections. It is important to monitor changes in the microbiology and epidemiology of *H. influenzae* as it can lead to clinical failure caused by antimicrobial resistance.

## Data availability statement

The raw data supporting the conclusions of this article will be made available by the authors, without undue reservation.

## Ethics statement

The studies involving human participants were reviewed and approved by Ethics committee of University of Antwerp (UAntwerpen) and the Antwerp University Hospital (UZA; ID 15/45/471 and ID 18/31/355). Written informed consent to participate in this study was provided by the participants’ legal guardian/next of kin.

## Author contributions

All authors listed have made a substantial, direct, and intellectual contribution to the work and approved it for publication.

## Funding

The study is supported by a research grant from Research Foundation Flanders (FWO Research Grant 1150017N, Antigoon ID 33341) and an investigator-initiated research grant from Pfizer.

## Conflict of interest

The authors declare that the research was conducted in the absence of any commercial or financial relationships that could be construed as a potential conflict of interest.

## Publisher’s note

All claims expressed in this article are solely those of the authors and do not necessarily represent those of their affiliated organizations, or those of the publisher, the editors and the reviewers. Any product that may be evaluated in this article, or claim that may be made by its manufacturer, is not guaranteed or endorsed by the publisher.
