# Supplementary material for: Haemophilus influenzae carriage and antibiotic resistance profile in Belgian infants over a three-year period (2016–2018)
Source: Front Microbiol. 2023 Apr 24;14:1160073. doi: 10.3389/fmicb.2023.1160073 (PMC10164969; doi:10.3389/fmicb.2023.1160073)
Supplement: Supplementary file 1 [file Data_Sheet_1.docx]

**Supplementary file**

Figure 1: Non-typeable and typeable of *Haemophilus influenzae* in the carriage (AOM and DCC) and invasive group per season*.*

Proportions per season of circulating serotypes in the carriage groups AOM (in orange) and DCC (in blue) as well as in the invasive group (in grey) detected in strains collected during the period 2016 – 2018 for the carriage group and 2015 – 2018 for the invasive group.

Figure 2: Biotypes of *Haemophilus influenzae* in the carriage (AOM and DCC) and invasive group per season*.*

Proportions per season of circulating biotypes in the carriage groups AOM (in orange) and DCC (in blue) as well as in the invasive group (in grey) detected in strains collected during the period 2016 – 2018 for the carriage group and 2015 – 2018 for the invasive group.
